# Response to cholinesterase inhibitors affects lifespan in Alzheimer’s disease

**DOI:** 10.1186/s12883-014-0173-4

**Published:** 2014-09-10

**Authors:** Carina Wattmo, Elisabet Londos, Lennart Minthon

**Affiliations:** Clinical Memory Research Unit, Department of Clinical Sciences, Malmö, Lund University, SE-205 02 Malmö, Sweden; Memory Clinic, Skåne University Hospital, SE-205 02 Malmö, Sweden

**Keywords:** Alzheimer’s disease, Cholinesterase inhibitors, Treatment effect, Life expectancy, Survival, Cognition, Activities of daily living, Predictors, Statistical models

## Abstract

**Background:**

A varying response to cholinesterase inhibitor (ChEI) treatment has been reported among patients with Alzheimer’s disease (AD). Whether the individual-specific response, specific ChEI agent or dose affects mortality is unclear. We aimed to examine the relationship between the 6-month response to ChEI and lifespan.

**Methods:**

Six hundred and eighty-one deceased patients with a clinical AD diagnosis and a Mini-Mental State Examination (MMSE) score of 10–26 at the start of ChEI therapy (baseline) were included in a prospective, observational, multicentre study in clinical practice. At baseline and after 6 months of treatment, the participants were assessed using the MMSE, the Alzheimer’s Disease Assessment Scale-cognitive subscale (ADAS-cog), the Clinician’s Interview-Based Impression of Change (CIBIC), the Instrumental Activities of Daily Living (IADL) scale, and the Physical Self-Maintenance Scale (PSMS). The individuals’ socio-demographic characteristics, ChEI dose, and date of death were recorded. Responses to ChEI and the association of possible risk factors with survival were analysed using general linear models.

**Results:**

A longer lifespan (mean of 0.5 years) was observed among the improved/unchanged patients, as measured by MMSE or CIBIC score, but not by ADAS-cog score, after 6 months of ChEI therapy. In the multivariate models, increased survival time was independently related to a better 6-month response in MMSE, CIBIC, IADL, and PSMS scores, female sex, no antihypertensive/cardiac or antidiabetic therapy, younger age, lower education, milder disease stage at baseline, and higher ChEI dose. Apolipoprotein E genotype did not affect mortality significantly. The patients who received a higher ChEI dose during the first 6 months had a mean lifespan after baseline that was 15 months longer than that of those who received a lower dose.

**Conclusions:**

A better short-term response to ChEI might prolong survival in naturalistic AD patients. In individuals who received and tolerated higher ChEI doses, a longer lifespan can be expected.

## Background

Alzheimer’s disease (AD) is a devastating neurodegenerative disorder characterized by progressive impairment of cognitive abilities, such as memory, orientation, language, and executive performance, and gradual loss of the capacity to carry out activities of daily living (ADL) [1]. A later consequence of AD is higher mortality compared with the nondemented elderly population. The disease represents the fifth leading cause of death in the USA in individuals aged ≥ 65 years [2]. The mean lifespan after the time of AD diagnosis varies between 3 and 10 years, depending on the patient’s age; however, individuals with AD can live considerably longer, up to 15–20 years [3-5]. Furthermore, the reduction of life expectancy compared with the general population ranges from 67% for patients diagnosed at 65 years of age to 39% for those diagnosed at 90 years of age [3].

Several studies have demonstrated a cholinergic deficit in AD [6,7], which became a target for therapeutic interventions. The “cholinergic hypothesis” led to the development of cholinesterase inhibitors (ChEIs), which are at present the main therapy used for mild-to-moderate AD. ChEIs prevent the degradation of acetylcholine (ACh) by the acetylcholinesterase enzyme, resulting in higher levels of ACh available in the synaptic cleft for receptor absorption. The treatment enhances the cholinergic transmission, thus improving the communication between neurons [8]. Multiple placebo-controlled randomised clinical trials have shown modest responses after 6 months of ChEI therapy regarding cognition, global performance, and ADL [9]. However, not every patient with AD benefits from ChEI treatment, because the level of response varies among individuals. In some AD studies, a more positive cognitive response to ChEI was observed in participants with a faster disease progression [10] or lower cognitive ability [11,12] and in patients taking larger doses of ChEIs [12,13]. Whether short-term response to ChEI alters the survival time in AD is not clear.

Many demographic and clinical factors decrease life expectancy in AD, such as male sex [14,15], older age [3,4,14,15], higher education [16], and greater cognitive impairment [14]. Moreover, cardiovascular disorders [14,17] and psychotic symptoms [18] might shorten lifespan in dementia. Because co-morbidity often accompanies AD, it might be difficult to point out a direct relationship between the disease and survival time. Some AD studies have investigated the association between ChEI therapy and mortality and reported conflicting results regarding whether these drugs increase the length of life [15,19]. Therefore, the influence of ChEIs and the complex impact of potential risk factors on survival warrant further investigation using multivariate statistical methods.

Expanded knowledge of the factors that affect mortality in AD, including the level of response to ChEI and drug dose, might provide a more accurate prognosis for the patients, which also represents essential information for their families and the clinicians. Moreover, a better understanding of predictors of survival in AD might be valuable for the community-based services, namely in the planning of care and of the economic resources available.

The aims of this study were 1) to examine the relationship between lifespan and the short-term response to ChEI treatment, 2) to investigate associations among survival time, drug agent and dosage of ChEI, and 3) to identify potential predictors that might influence these outcomes.

## Methods

### Study and subjects

The Swedish Alzheimer Treatment Study (SATS) was launched in 1997 to evaluate the long-term effectiveness of ChEI therapy (donepezil, rivastigmine, and galantamine) in AD patients in a routine clinical setting. SATS is a 3-year, open-label, observational, nonrandomised, multicentre study that has been described in earlier publications [12,20,21]. In total, 1,258 participants were prospectively enrolled from 14 memory clinics located in different geographical areas of Sweden. Among these, 880 individuals had baseline Mini-Mental State Examination (MMSE) [22] scores ranging from 10–26, indicating mild-to-moderate AD, and had fulfilled the 6-month post-baseline assessment. Up until December 31, 2012, 681 of these individuals (77%) had died and were included in the present study.

Outpatients who received the clinical diagnosis of dementia as defined by the Diagnostic and Statistical Manual of Mental Disorders, 4th edition (DSM-IV) [23] and of possible or probable AD according to the criteria of the National Institute of Neurological and Communicative Disorders and Stroke and the Alzheimer’s Disease and Related Disorders Association (NINCDS–ADRDA) [24] were considered for inclusion. The SATS participants were diagnosed by specialists in dementia disorders. Furthermore, the selected individuals had to be community-dwelling at diagnosis, had to have a responsible caregiver, and had to be assessable using the MMSE at the start of ChEI treatment (baseline). Medications other than ChEI were allowed, with the exception of memantine, and were documented at baseline.

All participants and/or caregivers gave their written informed consent to participate in the study, which was conducted according to the provisions of the Helsinki Declaration and was approved by the Ethics Committee of Lund University, Sweden.

The SATS patients were evaluated in a structured follow-up programme, which assessed cognition, global performance, instrumental and basic ADL, and community-based service utilisation immediately before the start of ChEI therapy, and then every 6 months during a 3-year period. The ChEI dose was recorded after 2 months of treatment and semiannually after baseline. Trained dementia nurses evaluated ADL capacity based on an interview with the caregiver. After inclusion and baseline assessments, the participants were prescribed ChEIs according to the approved product recommendations, as in routine clinical practice. The choice of drug agent and all decisions regarding dosage for each individual AD patient were left entirely to the discretion and professional judgment of dementia specialists. Most patients received an increased dose after 4–8 weeks of treatment, and we aimed at further dose increases depending on the chosen ChEI agent. However, for some individuals, the dose was reduced because of side effects. The responsible specialists and other staff at all participating SATS centres received joint, uniform training in Good Clinical Practice, in diagnostics, in usage of the rating scales and regarding the performance of the evaluations and the study. In addition, research nurses from the main centre (Memory Clinic, Malmö) supervised the SATS via careful monitoring and personal visits to the various centres throughout the entire study.

### Outcome measures

Cognitive status was assessed using the MMSE scale, which ranges from 0 to 30 and in which a lower score indicates more impaired cognition, and the Alzheimer’s Disease Assessment Scale-cognitive subscale (ADAS-cog) (0–70 points) [25], in which a lower score indicates higher cognitive ability. The Clinician Interview-Based Impression of Change (CIBIC) [26] was used as a global measure of “change from the baseline.” The evaluations of change in global performance from the start of ChEI treatment were performed at all intervals using a 7-point scale that varied from 1 (very much improved) to 7 (marked worsening), with 4 indicating no change. No guidelines or descriptors were provided to define the individual ratings. The classification between, e.g., minimally improved or very much improved was left to the physician’s clinical judgment.

The capacity to perform daily activities was assessed using the Instrumental Activities of Daily Living (IADL) scale [27], which comprises eight items: ability to use the telephone, shopping, food preparation, housekeeping, ability to do laundry, mode of transportation, responsibility for own medications, and ability to handle finances. Each item was scored from 1 (no impairment) to 3–5 (severe impairment), thus allowing a total range of 8–31 points. Some of the instrumental tasks may be gender-dependent among elderly persons. Therefore, a mathematical correction of the sum of the IADL scores was performed to prevent a bias in the results from those tasks. The equation used the data from the rated items to estimate a total score within the range of the total IADL scale [28]. The Physical Self-Maintenance Scale (PSMS) [27] comprises six items: toilet, feeding, dressing, grooming, physical ambulation, and bathing. Each item was scored from 1 (no impairment) to 5 (severe impairment), thus allowing a total range of 6–30 points.

Using the 12-digit personal identity number assigned to each resident of Sweden, all SATS patients were investigated with the help of the Swedish population register (Swedish Tax Agency) regarding whether they were still alive on December 31, 2012. If not, the date of death was recorded.

### Statistical analyses

The Statistical Package for Social Sciences (SPSS) software (version 21.0; IBM Corporation, Armonk, NY, USA) was used to perform the statistical analyses. The level of significance was defined as *P* < 0.05, unless otherwise specified, and all tests were two-tailed. Parametric tests were used because of the large sample size and the approximately normally distributed continuous potential predictors. Independent-sample *t* tests were used to compare the differences between the means for two groups, and *χ*^2^ tests were computed for analyses of categorical variables.

Kaplan–Meier graphs were used to illustrate the differences in time to death in the figures: “improved/unchanged vs deteriorated in MMSE score” and “high vs low ChEI dose”. The distribution of time was compared using the log-rank test.

### General linear models

The multivariate approach of general linear models was used because of the large sample size of deceased participants for whom all data were available, including the date of death. The relationship between potential predictors, including the response to ChEI therapy after 6 months in each model (MMSE, ADAS-cog, CIBIC, IADL, or PSMS scale), and survival time was investigated. The dependent normally distributed variable was the length of life (in years) after the start of ChEI treatment. Based on previous knowledge of risk factors of life expectancy in AD, several socio-demographic and clinical characteristics were included in each of the above-mentioned models. The independent variables were: age at baseline; clinician’s estimate of age at onset; sex; years of education; number of apolipoprotein E (APOE) ε4 alleles; solitary living; cognitive, instrumental, and basic ADL abilities at baseline (or global rating in the CIBIC model); number of concomitant medications; specific medications used (antihypertensive/cardiac therapy, antidiabetic drugs, lipid-lowering agents, estrogens, nonsteroidal anti-inflammatory drugs (NSAIDs)/acetylsalicylic acid, antidepressants, antipsychotics, and anxiolytics/sedatives/hypnotics); type of ChEI agent; drug dose; and the 6-month response to ChEI measured by the MMSE, ADAS-cog, CIBIC, IADL, or PSMS score. Nonsignificant variables (*P* > 0.05) were removed in a backward stepwise elimination manner.

The ChEI dose could vary during the treatment period for an individual patient and among patients. Therefore, the mean dose used during the first 6 months of therapy was calculated for each individual. Furthermore, to obtain a similar metric of percent maximum dosage for the three ChEI agents, the mean dose was divided by the maximum recommended dose for each drug agent, i.e., 10 mg of donepezil, 12 mg of rivastigmine (oral therapy), and 24 mg of galantamine.

Response was calculated as the change in score between the 6-month assessment after the start of ChEI treatment and the baseline for each scale (MMSE, ADAS-cog, IADL, or PSMS). To facilitate comparisons between the scales, changes in the scores calculated as positive values should be interpreted as indicating improvement, and those calculated as negative values interpreted as indicating decline. The evaluations of change in global performance (CIBIC) after 6 months were scored as 1–3 (improved), 4 (unchanged), and 5–7 (worsened).

## Results

The socio-demographic and clinical characteristics of the 681 deceased AD patients are shown in Table 1. Their lifespan after the start of ChEI therapy (baseline) was 5.83 ± 2.76 years (mean ± standard deviation (SD)). Women had a significantly longer survival (6.25 ± 2.83 vs 5.13 ± 2.48 years; t (679) = −5.43; *P* < 0.001).

**Table 1:** **Socio-demographic and clinical characteristics (** ***n*** **= 681)**

**Variable**	
Female sex	423 (62%)
APOE ε4 carrier, (*n* = 667)	437 (66%)
Solitary living at baseline	240 (35%)
Antihypertensives/cardiac therapy	289 (42%)
Antidiabetics	30 (4%)
Lipid-lowering agents	63 (9%)
Estrogens	51 (7%)
NSAIDs/acetylsalicylic acid	212 (31%)
Antidepressants	174 (26%)
Antipsychotics	30 (4%)
Anxiolytics/sedatives/hypnotics	92 (14%)
Variable	Mean ± standard deviation
Estimated age at onset (years)	73.0 ± 6.9
Estimated duration of AD at baseline (years)	3.1 ± 2.0
Age at first assessment (years)	76.1 ± 6.5
Education (years)	9.3 ± 2.4
Age at death (years)	81.9 ± 6.6
MMSE score at baseline	20.9 ± 3.9
ADAS-cog score (0–70) at baseline	22.0 ± 9.1
IADL score at baseline	16.6 ± 5.4
PSMS score at baseline	7.7 ± 2.3
Number of concomitant medications at baseline	3.0 ± 2.5

*Abbreviations*: *ADAS-cog* Alzheimer’s Disease Assessment Scale-cognitive subscale, *APOE* apolipoprotein E, *IADL* Instrumental Activities of Daily Living scale, *MMSE* Mini-Mental State Examination, *NSAIDs* nonsteroidal anti-inflammatory drugs; PSMS, Physical Self-Maintenance Scale.

### Response after 6 months of ChEI treatment

General linear models using the time to death after the start of ChEI therapy as the dependent variable were built to identify the socio-demographic and clinical factors that affected the life expectancy of AD patients. The multivariate models and significant predictors are presented in Tables 2 and 3. A longer time to death was associated with a more positive response in cognitive (MMSE model, but not ADAS-cog model) or functional capacity after 6 months of ChEI treatment, female sex, no antihypertensive/cardiac or antidiabetic therapy, younger age, lower level of education, better cognitive and ADL abilities at the baseline, and higher dose of ChEI. Regarding the CIBIC model, a longer lifespan was related to no worsening in global performance after 6 months of ChEI therapy, female sex, no antihypertensive/cardiac or antidiabetic therapy, younger age, a better global rating at the baseline, and a higher dose of ChEI (Table 2).

**Table 2:** **Factors that affected the lifespan of AD patients after the start of ChEI treatment (final general linear cognitive and global models)**

	***MMSE***		***ADAS-cog***		***CIBIC***	
	**R = 0.399, R** ^**2**^ **= 0.159,** ***P*** **< 0.001**		**R = 0.405, R** ^**2**^ **= 0.164,** ***P*** **< 0.001**		**R = 0.365, R** ^**2**^ **= 0.133,** ***P*** **< 0.001**	
Significant predictors	β (95% CI)	*P* value	β (95% CI)	*P* value	β (95% CI)	*P* value
Intercept	7.782 (4.742, 10.823)	<0.001	9.796 (6.880, 12.712)	<0.001	9.848 (6.994, 12.702)	<0.001
MMSE or ADAS-cog score at baseline	0.066 (0.008, 0.124)	0.025	−0.031 (−0.055, −0.007)	0.013	na	
Change in MMSE or ADAS-cog score after 6 months of ChEI therapy^a^	0.072 (0.009, 0.135)	0.025		ns	na	
CIBIC score at baseline	na		na		−0.458 (−0.722, −0.195)	0.001
Worsening in CIBIC (score 5–7) after 6 months of ChEI therapy (no = 0, yes = 1)	na		na		−0.502 (−1.001, −0.003)	0.048
Sex (male = 0, female = 1)	0.888 (0.473, 1.302)	<0.001	0.886 (0.467, 1.304)	<0.001	1.029 (0.614, 1.443)	<0.001
Antihypertensive/cardiac therapy (no = 0, yes = 1)	−0.646 (−1.053, −0.239)	0.002	−0.663 (−1.075, −0.252)	0.002	−0.512 (−0.924, −0.100)	0.015
Antidiabetics (no = 0, yes = 1)	−1.563 (−2.537, −0.588)	0.002	−1.627 (−2.607, −0.646)	0.001	−1.614 (−2.578, −0.650)	0.001
Age at first assessment (years)	−0.052 (−0.084, −0.019)	0.002	−0.051 (−0.084, −0.019)	0.002	−0.062 (−0.093, −0.031)	<0.001
Education (years)	−0.104 (−0.189, −0.019)	0.016	−0.104 (−0.189, −0.020)	0.015		ns
IADL score at baseline	−0.070 (−0.113, −0.027)	0.001	−0.074 (−0.117, −0.031)	0.001	na	
ChEI dose^b^	0.021 (0.009, 0.033)	<0.001	0.023 (0.011, 0.034)	<0.001	0.022 (0.010, 0.034)	<0.001

Apolipoprotein E genotype, solitary living, age at onset, basic ADL ability, number of medications and specific concomitant medications (lipid-lowering agents, estrogens, nonsteroidal anti-inflammatory drugs/acetylsalicylic acid, antidepressants, antipsychotics, and anxiolytics/sedatives/hypnotics) used at the start of ChEI treatment (baseline) were not significant.

β values were unstandardized and are expressed per 1 unit increase for continuous variables, and for the condition present for dichotomous variables.

^a^For clarity, clinical improvements for all scales were calculated as positive changes from baseline.

^b^Mean percentage of the maximum recommended dose during the first 6 months of ChEI therapy, i.e., 10 mg for donepezil, 12 mg for rivastigmine, and 24 mg for galantamine.

*Abbreviations*: *ADAS-cog* Alzheimer’s Disease Assessment Scale-cognitive subscale, *ChEI* cholinesterase inhibitors, *CI* confidence interval, *CIBIC* Clinician’s Interview-Based Impression of Change, *IADL* Instrumental Activities of Daily Living, *MMSE* Mini-Mental State Examination, *na* not applicable, *ns* not significant.

**Table 3:** **Factors that affected the lifespan of AD patients after the start of ChEI treatment (final general linear functional models)**

	**IADL**		**PSMS**	
	**R = 0.397, R** ^**2**^ **= 0.158,** ***P*** **< 0.001**		**R = 0.394, R** ^**2**^ **= 0.155,** ***P*** **< 0.001**	
Significant predictors	β (95% CI)	*P* value	β (95% CI)	*P* value
Intercept	9.402 (6.526, 12.278)	<0.001	8.430 (5.377, 11.483)	<0.001
IADL or PSMS score at baseline	−0.112 (−0.151, −0.073)	<0.001	−0.170 (−0.262, −0.077)	<0.001
Change in IADL or PSMS score after 6 months of ChEI therapy^a^	0.094 (0.026, 0.162)	0.007	0.151 (0.036, 0.267)	0.010
Sex (male = 0, female = 1)	0.910 (0.494, 1.326)	<0.001	1.008 (0.593, 1.424)	<0.001
Antihypertensive/cardiac therapy (no = 0, yes = 1)	−0.611 (−1.021, −0.201)	0.004	−0.551 (−0.963, −0.140)	0.009
Antidiabetics (no = 0, yes = 1)	−1.543 (−2.536, −0.551)	0.002	−1.672 (−2.669, −0.675)	0.001
Age at first assessment (years)	−0.043 (−0.075, −0.011)	0.009	−0.053 (−0.085, −0.021)	0.001
Education (years)	−0.090 (−0.174, −0.006)	0.036	−0.098 (−0.183, −0.013)	0.024
MMSE score at baseline		ns	0.061 (0.006, 0.115)	0.030
ChEI dose^b^	0.019 (0.008, 0.031)	0.001	0.017 (0.005, 0.029)	0.005

Apolipoprotein E genotype, solitary living, age at onset, number of medications and specific concomitant medications (lipid-lowering agents, estrogens, nonsteroidal anti-inflammatory drugs/acetylsalicylic acid, antidepressants, antipsychotics, and anxiolytics/sedatives/hypnotics) used at the start of ChEI treatment (baseline) were not significant.

β values were unstandardized and are expressed per 1 unit increase for continuous variables, and for the condition present for dichotomous variables.

^a^For clarity, clinical improvements for all scales were calculated as positive changes from baseline.

^b^Mean percentage of the maximum recommended dose during the first 6 months of ChEI therapy, i.e., 10 mg for donepezil, 12 mg for rivastigmine, and 24 mg for galantamine.

*Abbreviations*: *ChEI* cholinesterase inhibitors, *CI* confidence interval, *IADL* Instrumental Activities of Daily Living, *MMSE* Mini-Mental State Examination, *ns* not significant, *PSMS* Physical Self-Maintenance Scale.

**Figure 1:**
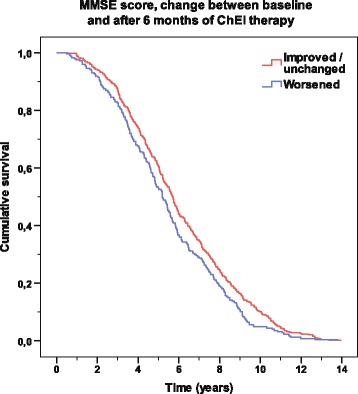
**Time to death according to response in MMSE score.** Kaplan–Meier graph of the distribution of time from the start of cholinesterase inhibitor treatment to death for the variable “improved/unchanged (≥0 point change) vs deteriorated (<0 point change)” based on the Mini-Mental State Examination (MMSE) score after 6 months. A log-rank test showed a significant difference between the two groups (*P* = 0.013).

As an example, 426 SATS participants (63%) showed improvement/no change (≥0 point change) in MMSE score after 6 months of ChEI treatment. Among them, 161 individuals (24%) exhibited improvement, i.e., had an increase of 3 or more MMSE points. The improved/unchanged MMSE group exhibited a longer lifespan after the baseline than did the deteriorated group (6.03 ± 2.78 vs 5.48 ± 2.64 years; t (671) = 2.52; *P* = 0.012). No significant difference was detected between the improved and unchanged AD patients. Figure 1 displays the Kaplan–Meier graph of the improved/unchanged vs deteriorated groups, as measured by MMSE score (*P* = 0.013). No differences in sex, antihypertensive/cardiac therapy, antidiabetics, age at baseline, years of education, MMSE score at baseline, or ChEI dose were observed between the two groups.

Regarding the ADAS-cog score, 333 patients (49%) showed improvement/no change (≤0 point change) after 6 months of ChEI therapy. Among them, 167 individuals (25%) exhibited improvement, i.e., had a decrease of at least 4 ADAS-cog points. The 6-month change in ADAS-cog score showed no significant association with survival time.

General linear multivariate modeling revealed that a worsening in CIBIC (score, 5–7) after 6 months of ChEI treatment implied, on average, a shorter life expectancy (by 0.5 years) (Table 2). Global improvement (CIBIC score, 1–3) after 6 months was observed in 221 patients (33%), no change (CIBIC score, 4) in 279 patients (41%), and worsening in 175 patients (26%).

Regarding change in IADL or PSMS score, a respective mean shorter lifespan of 1.1 or 1.8 months per point of deterioration after 6 months of ChEI therapy was found (Table 3). The 6-month functional decline was on average 1.35 ± 2.96 points in IADL score and 0.40 ± 1.78 points in PSMS score.

### ChEI treatment

Among the 681 AD patients, 406 (60%) received donepezil, 137 (20%) rivastigmine, and 138 (20%) galantamine. During the first 6 months of ChEI therapy, the mean ± SD doses of donepezil, rivastigmine, and galantamine were 6.2 ± 1.6, 4.8 ± 1.1, and 11.8 ± 3.1 mg, respectively. No significantly different effect on lifespan was found among the three drug agents after adjusting for age at the baseline and the interaction effect of type of ChEI with age.

**Figure 2:**
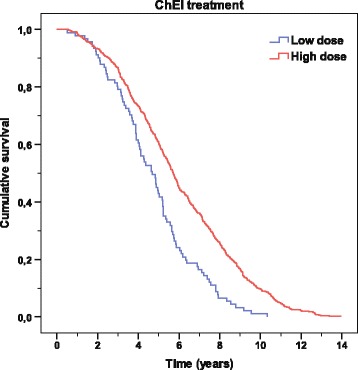
**Time to death according to ChEI dose.** Kaplan–Meier graph of the distribution of time from the start of cholinesterase inhibitor (ChEI) treatment to death for the variable “ChEI dose”. A log-rank test showed a significant difference between high vs low doses during the first 6 months of therapy (*P* < 0.001). The cutoff median values for the drug doses were 5.0 mg for donepezil, 5.0 mg for rivastigmine, and 13.3 mg for galantamine.

The individuals who received a higher dose of ChEI during the first 6 months, regardless of drug agent, exhibited a longer mean survival time after the start of treatment than did those receiving a lower dose (6.02 ± 2.82 vs 4.76 ± 2.09 years; t (671) = −4.08; *P* < 0.001). The Kaplan–Meier analysis also showed a significant difference in lifespan between patients receiving a low or a high dose of ChEI (*P* < 0.001; Figure 2). The median cutoff values for the dose were 5.0 mg for donepezil, 5.0 mg for rivastigmine, and 13.3 mg for galantamine. No significant differences in sex, antihypertensive/cardiac therapy, antidiabetics, age at baseline, years of education, and cognitive or functional scores at baseline were detected between the two groups.

## Discussion

This observational AD study performed in clinical practice showed that a longer lifespan was independently related to a more favourable response in cognitive (MMSE model only), global, or functional ability after 6 months of ChEI therapy; female sex; no antihypertensive/cardiac or antidiabetic therapy; younger age; a lower level of education (not the CIBIC model); milder disease severity at the baseline; and higher dose of ChEI. APOE genotype did not affect mortality significantly. The patients who received a higher ChEI dose during the first 6 months had a mean survival time after the baseline that was more than 1 year longer than that of those receiving a lower dose.

AD reduces life expectancy in untreated individuals, particularly in those affected at younger ages [3]. Whether ChEI treatment has a beneficial or detrimental effect on mortality is unknown, as the few AD studies that have analysed this association have reported conflicting results [15,19]. In this study, the mean survival of 5.83 years after the time of AD diagnosis (approximately the start of ChEI therapy) was similar to that of untreated patients [3,14]. Rountree et al. [15] found that anti-dementia drug exposure was not significantly related to mortality in a community-based AD cohort that was followed for a mean period of 3 years. In a large-sample study of nursing-home residents with dementia, Gasper et al. observed that the survival rate after 2 years was higher in the ChEI-treated group compared with the untreated group. Nevertheless, individuals who are treated with ChEIs attend regular visits to their physician, and could also be receiving more aggressive pharmacological therapies against other co-morbid disorders, which might increase their length of life [19]. Both of the studies mentioned above included adjustment for a broad range of covariates, such as socio-demographic characteristics, dementia severity and major co-morbid illnesses. The patients with AD in the community-based cohort [15] were 10 years younger, on average, than the nursing-home residents [19] (73 vs 83 years). A recent study from our group that compared the SATS participants with untreated AD cohorts found no difference between these groups in patients aged < 85 years; however, a longer lifespan was observed among the ChEI-treated oldest old participants [5]. Older individuals may have less hereditary and aggressive forms of AD. In addition, their cognitive reserve capacity may be reduced, which could lead to the detection of the disease, diagnosis and ChEI therapy at an earlier stage. In another study from the SATS, older age was reported as an independent predictor of better cognitive short-term response to ChEI and longitudinal outcome [12]. These factors might imply a lower mortality rate in the oldest AD patients.

In the current study, the improved/unchanged participants, as measured by MMSE score after 6 months of ChEI treatment, exhibited a lifespan that was 0.5 years longer on average compared with the deteriorated group. Similarly, a worsening in the global CIBIC score suggested a 6-month shortening of life expectancy in AD. Individuals who demonstrated functional response, particularly in basic ADL, also had a significantly longer survival. No difference in mortality among the responder groups was observed when using the ADAS-cog scale. The ADAS-cog is a more complex cognitive measure than the MMSE test, and it includes a greater number of items. The proportion of patients who were categorized as improved/unchanged according to ADAS-cog was smaller compared with that categorized using the MMSE scale (49% vs 63%), which might be one explanation for this inconsistent result. Randomised clinical trials have demonstrated that ChEIs might delay both cognitive decline and loss of ADL [29-31]. A modest post-treatment improvement in cognitive rating scales for about 6–12 months before the score returned to baseline value was reported by previous AD studies [32-34]. Individuals who responded better to ChEI cognitively exhibited a lower cognitive ability at the baseline in several [11,12,31] but not all [35] studies. However, a subsequent faster deterioration over time was observed in the patients who were more severely impaired after their initial response to therapy [12]. A greater reversible cholinergic deficit in the more advanced stages of AD is a possible explanation for the finding mentioned above, suggesting that this subpopulation is more responsive to ChEI [36]. Another explanation might be that the change in the score on a certain assessment scale is expected to be larger at the level of function, e.g., moderate AD, at which the scale measures the person’s abilities most accurately [37]. Articles stemming from the SATS reported that a higher cognitive status at the baseline was associated with better longitudinal outcome in cognition [12], and that individuals who initially exhibited a more positive functional response to ChEI therapy were less cognitively impaired at the baseline and showed a significantly higher ADL capacity after 3 years of treatment [38]. Therefore, the participants’ cognitive and functional scores were included in our general linear models as independent predictors. Nevertheless, the responders exhibited a significantly longer survival. The above-mentioned findings indicate that response to ChEI, as measured using different scales, prolongs life by a few months in AD patients. A more preserved cognitive status at the start of therapy can lead to a better ability to maintain higher levels of cognitive and ADL performance over longer periods, which underscores the importance of initiation of anti-dementia drugs at an early stage of the disease. The effects of initial response to ChEI and the subsequent post-treatment delays in symptom progression suggest that the observed mean 6-month increase in lifespan occurs at a higher cognitive and functional level and not in the later, more advanced stages of AD.

Here, concomitant antihypertensive/cardiac therapy and antidiabetic therapy reduced the mean survival time by 6–8 months and ~1.5 years, respectively. Consistently, co-morbid disorders, particularly cardio/cerebrovascular and respiratory diseases, and diabetes, increase mortality in most dementia studies [14,17,39] and in the general population [40]. Because concomitant illnesses are commonly observed in elderly persons, it is difficult to investigate direct associations between AD and lifespan. In the present study, we adjusted the multivariate models for usage of different types of medications. Nevertheless, other factors related to co-morbidity might affect survival time in AD, such as severity and type of somatic disorders or psychiatric symptoms, which were not addressed by the variables included in the models. Moreover, a shorter life expectancy among males and older individuals was observed in this study and in most previous AD studies [14,15], and in the general population [40]. Male sex, older age, and antihypertensive/cardiac/antidiabetic therapy were independent predictors of higher mortality in all of our statistical models, regardless of the scales used, which demonstrates the accuracy and consistency of the results.

The current study of ChEI-treated patients with AD showed that those with a higher education level have a shorter lifespan, similar to the findings of Stern et al. regarding untreated individuals [16]. However, most previous studies have demonstrated that the individuals’ level of education does not affect life expectancy [3,14,15]. A faster cognitive decline among patients with a higher level of education has been reported in several [12,41,42] but not all [43,44] ChEI-treated and untreated AD cohorts. Furthermore, a greater proportion of APOE ε4 carriers among those with a higher education level has also been observed [12]. In studies from the USA [3,14,15,44], the majority of participants are highly educated, which might imply that the variance in the cohorts is too small to detect possible differences. Individuals with a high education level were assumed to have a more pronounced cognitive reserve and, therefore, would be less vulnerable to the effects of neurodegeneration. This might lead to better performance on cognitive tests and, consequently, later detection of the disease [41]. A higher level of education can be a risk factor for worse prognosis in AD, suggesting a more advanced disease at the manifestation of clinical symptoms and at treatment onset, a faster progression rate, and a shorter survival.

In the present study, the patients who received and tolerated a higher dose of ChEI, regardless of drug agent, exhibited a mean of 15 months of extension in lifespan. Higher doses have previously been related to more positive long-term cognitive and functional outcomes [12,13,21]. Moreover, a significant association between a higher dose of ChEI and a lower amount of home help services [45], and delays in the need for nursing home placement [28,46], has been reported. These results show that a higher ChEI dose would be beneficial as long as the individuals can tolerate this medication. The slowing of disease progression and the postponed need of community-based services indicate that the possible increase in life expectancy occurs in a less advanced stage, when the AD patients are able to live in their own homes.

The advantages of the SATS are the 6-month, prospective, well-structured cognitive, global, and functional assessments after the start of ChEI therapy in a large AD cohort with co-morbidities and concomitant medications enrolled from memory clinics located across Sweden. Access to the Swedish health care system is publicly funded for residents; thus, not dependent on the patient’s income or health insurance coverage [47]. This leads to the assumption that the SATS participants are representative of the general population. A limitation of the SATS is that, as in other long-term naturalistic studies, it is not placebo controlled because of ethical considerations, or randomised according to drug agent. Clinicians who are specialised in dementia disorders decided on the type of ChEI and the dose for each individual patient, in accordance with the standard routine in clinical practice. Another shortcoming of the study was that the clinical diagnosis of AD was not confirmed via autopsy. Additional factors that were not investigated in the SATS might also influence mortality. For example, a recent study reported by our group found that cerebral inflammation independently predicted an early death in AD. The effect of ChEI was not addressed in that study [48]. Furthermore, ChEI therapy could entail wide-spread effects beyond the central nervous system that might affect lifespan. ACh synthesis has been detected in various types of immune cells, muscle cells and epithelial cells (e.g., of the airways and of the alimentary and urogenital tracts) [49]. A review of the role of ChEIs in the modulation of the immune response reported that these drug agents might influence the immune system [50].

This is the first AD study to analyse the association between short-term response to ChEI therapy and survival. Additional studies are warranted to confirm our findings. Regardless of whether response to ChEIs prolongs life, other aspects that were not evaluated in the SATS, such as the patients’ quality of life and the caregiver burden during this extended time, need to be investigated. An increased understanding of the impact of ChEI treatment on life expectancy is important for the estimation of the patients’ prognosis by the treating physicians. Detailed information regarding factors that affect survival time in AD might be a valuable tool for the health authorities to investigate the effects and costs of the disease from a societal perspective. Knowledge of the potential effects of ChEIs on lifespan is necessary for the assessment of the effect of future disease-modifying therapies on mortality in AD.

## Conclusions

In conclusion, the current study of response to ChEI after 6 months of treatment showed a few months prolonged lifespan among the improved/unchanged AD patients regarding cognition (MMSE score, but not ADAS-cog score), global performance, and functional capacity. A shorter survival time was observed in men, older individuals, and in those taking antihypertensive/cardiac or antidiabetic medications. These findings are consistent with those of other dementia studies and for the general population, thus showing consistency in our models. A higher level of education was also related to shorter life expectancy, which might reflect a more advanced disease at the point of seeking care and consequently a more rapid AD progression. The patients who could tolerate a higher dose of ChEI, regardless of drug agent, during the first 6 months of therapy exhibited a longer mean lifespan (by more than 1 year) than did those receiving a lower dose. Earlier reports of post-treatment delays in disease progression and a decrease in community-based service utilisation, lead to the assumption that this increase in the length of life occurs at a higher cognitive and functional level, with no increase in survival during the most debilitated stages of AD.

## Abbreviations

ACh: Acetylcholine
AD: Alzheimer’s disease
ADAS-cog: Alzheimer’s disease assessment scale - cognitive subscale
ADL: Activities of daily living
APOE: Apolipoprotein E
ChEI: Cholinesterase inhibitors
CI: Confidence interval
CIBIC: Clinician interview-based impression of change
IADL: Instrumental activities of daily living scale
MMSE: Mini-mental state examination
NSAIDs: Non-steroidal anti-inflammatory drugs
PSMS: Physical self-maintenance scale
SATS: Swedish alzheimer treatment study
SD: Standard deviation
